# CD86 Molecule Might Be a Novel Immune-Related Prognostic Biomarker for Patients With Bladder Cancer by Bioinformatics and Experimental Assays

**DOI:** 10.3389/fonc.2021.679851

**Published:** 2021-08-06

**Authors:** Xin Yan, Guo-Wei Du, Zhao Chen, Tong-Zu Liu, Sheng Li

**Affiliations:** ^1^Department of Urology, Zhongnan Hospital of Wuhan University, Wuhan, China; ^2^Department of Biological Repositories, Zhongnan Hospital of Wuhan University, Wuhan, China

**Keywords:** bladder cancer, immune-related genes, weighted gene co-expression network analysis (WGCNA), immune cell infiltration, prognosis

## Abstract

As one of the most common malignancies in the urinary system, bladder cancer (BC) occupies a high mortality and recurrence rate. BC carries an ominous prognosis. Thus, we aimed to identify a novel immune-related prognostic biomarker and therapeutic target for immunotherapy in the present study. We first constructed a co-expression network based on immune-related genes (IRGs). Two key modules showed high association with the clinical feature interested us most were further identified. Forty-five IRGs were screened out and regarded as hub genes in the co-expression network. We further constructed a protein-protein interaction (PPI) network, and five independent methods were used for hub gene identification. Three hub genes were identified in the present study. CD86 molecule (CD86) was screened out by performing overall survival (OS) analysis. Subsequent analyses by using some bioinformatics and experimental assays confirmed that CD86 was an immune-related prognostic biomarker, which might be a novel target for immunotherapy in BC. A small molecule drug named *suloctidil* was also identified, which showed potential for BC treatment.

## Introduction

Bladder cancer (BC) is the most common malignant tumor in the urinary system (1). In 2018, about 550,000 new cases were diagnosed worldwide and about 200,000 patients died according to recent statistics from the International Agency for Research on Cancer (IARC), part of the World Health Organization (WHO) (2). At present, transurethral resection of bladder tumor is the main method for the treatment of BC (3). However, in all tumors, BC shows a very high recurrence rate (30–70%) and often progresses to more aggressive forms of BC (4). The 5-year survival rate of BC is only 15%, which means most patients with BC have to face poor prognosis (5).

In recent years, immunotherapy has been used in a variety of tumors, such as clear cell renal cell carcinoma (6), breast cancer (7), and lung cancer (8). Clinical studies have shown that bladder cancer (BC) is immunogenic (9). Intravesical instillation of bacillus Calmette-Guerin (BCG) is the most commonly used immunotherapy for bladder cancer, but 25% of patients still do not respond to BCG (10, 11). Checkpoint inhibition immunotherapy has also been applied to the treatment of BC, but only 25% advanced/metastatic bladder cancers respond to anti-programmed cell death protein 1 (PD-1)/programmed cell death 1 ligand 1 (PD-L1) immune checkpoint blockade (ICB) (12). Therefore, screening out an immune-related prognostic biomarker, which might be a more accurate and comprehensive target for immunotherapy, is badly needed.

For the first time, we constructed a co-expression network based on immune-related genes (IRGs) in BC by applying Weighted Gene Co-Expression Network Analysis (WGCNA) (13) [WGCNA is a widely used method in large gene expression data analysis and gene module associated with clinical feature identification in present (14, 15)]. Relying on this method, we screened out some potential prognostic biomarkers in clear cell renal cell carcinoma (16) and acute myeloid leukemia (17). In this study, a total 45 IRGs were screened out after WGCNA. Finally, CD86 molecule (CD86) was identified by using several bioinformatics and experimental assays and regarded as an immune-related prognostic biomarker in BC, which had great effects for assessing prognosis of BC patients and might be a novel target for immunotherapy.

## Materials And Methods

### BC Data Collection

GSE32548 (18) performed on GPL6947 was downloaded from Gene Expression Omnibus (GEO) database (http://www.ncbi.nlm.nih.gov/geo/) (19, 20), which included 128 BCs with complete clinical information. In this study, we constructed co-expression network based on this data set. Another GEO data set GSE13507 (21, 22) was also downloaded from this database for validation of our findings, performed on GPL6102, which had 165 BCs with clinical information. The Cancer Genome Atlas (TCGA) database (https://genome-cancer.ucsc.edu/) (23) characterized over 20,000 primary cancer and matched normal samples spanning almost all the cancer types. Thus, we retrieved BC microarray data from this database. After excluding unqualified samples, a total of 408 BC samples with complete clinical information were used for validation in this study.

### Data Preprocessing

**Figure 1:**
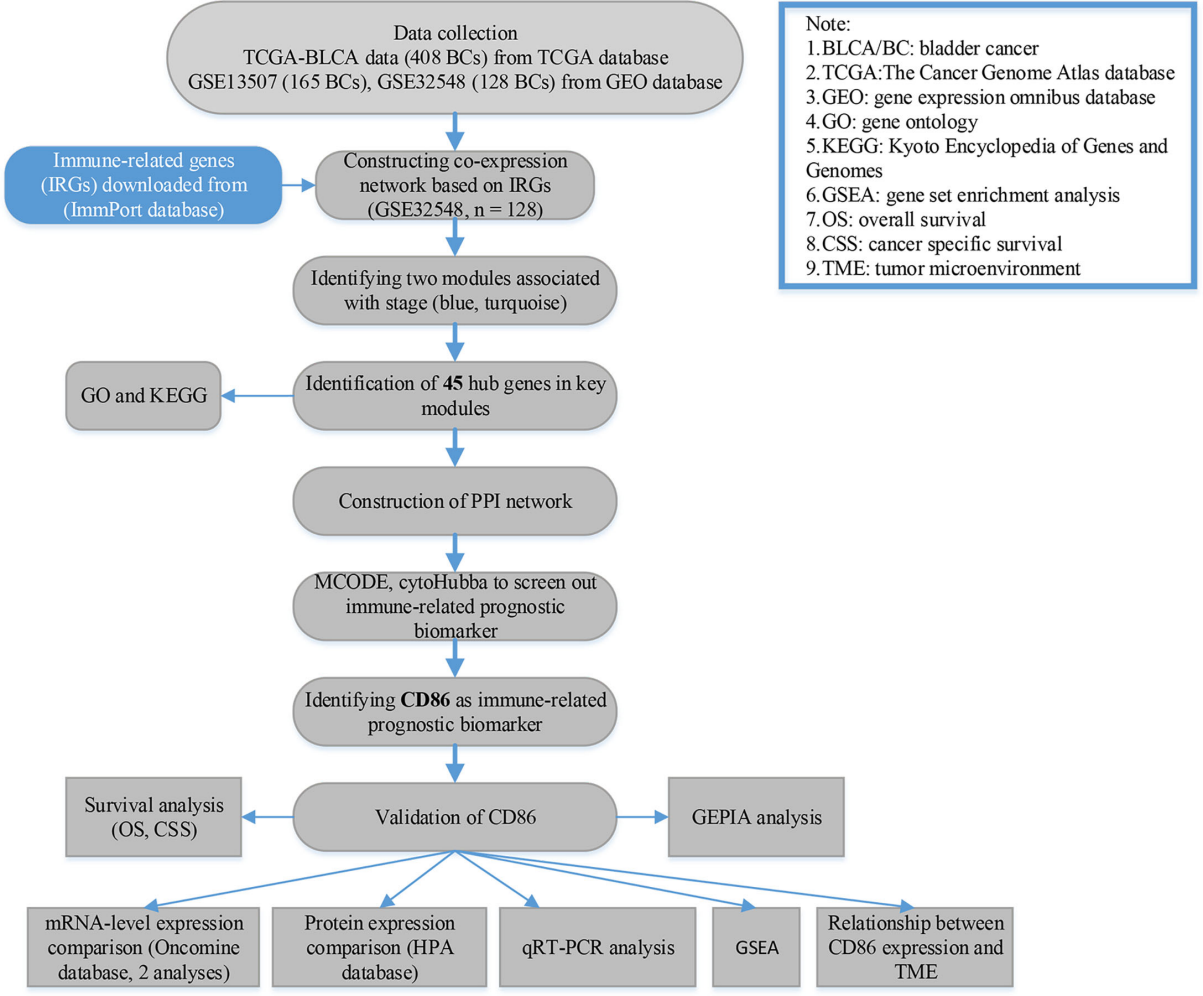
Flow diagram of data preparation, processing, analysis, and validation in this study.

For GSE32548 and GSE13507, we first downloaded the raw expression data and then normalized and transformed the data by using R package “affy” (24). As for TCGA-bladder urothelial carcinoma (BLCA) data displayed as counts number, normalization and log2 transformation were conducted by using package “DEseq.2” (25) in R software. The flow diagram of the present study is shown in **Figure 1** in detail.

### Co-Expression Network Construction

We first downloaded a comprehensive list of immune-related genes (IRGs) from the ImmPort database (https://immport.niaid.nih.gov) (26), which included 2,498 IRGs. Then 1,333 genes overlapped between IRGs, and genes in GSE32548 were included for the WGCNA. Two independent methods, including gsg (goodSamplesGenes) method and sample network method, were used for outlying sample identification of the expression profile of the 1333 genes. Only samples with Z.Ku ≥ −2.5 were used to construct a co-expression network by using R package “WGCNA” (samples were regarded as outliers when Z.Ku < −2.5, which were removed from the expression profile). Soft threshold power beta (β) was chosen by using the scale-free topology criterion (27, 28). In this study, genes were classified into gene modules by using the dynamic hybrid branch cutting method. Parameters for branch splitting were set as follows: minClusterSize = 30, and deepSplit = 2. In this study, we created a cluster tree of module eigengenes (MEs) to merge some highly related modules. Modules will be merged if their pairwise correlation was larger than 0.75. Moreover, a multidimensional scaling (MDS) plot was plotted to estimate the bio-similarity of the modules.

### Key Module Screening

After gene module identification, we further screen out key module related to the trait (pathological stage), which interested us most. The correlation between genes and trait was quantified by calculating gene significance (GS). Module significance (MS) was further defined based on GS. Moreover, the relationship between ME and gene expression matrix was quantified by calculating module membership (MM). Finally, the module most positively correlated with pathological stage and the module most negatively related to pathological stage were considered as key modules in the present study.

### Function and Pathway Enrichment Analysis

First, we screened hub genes in key modules by measuring cor.geneModuleMembership and cor.geneTraitSignificance. In this study, only genes with |cor.geneModuleMembership| > 0.80 and |cor.geneTraitSignificance| > 0.20 were regarded as hub genes in the modules. By conducting Gene Ontology (GO) (29) enrichment analysis and Kyoto encyclopedia of Genes and Genomes (KEGG) (30) pathway analysis, we could understand biological meaning behind hub genes in key modules. Both the two analyses were identified based on R package “clusterProfiler” (31). Gene sets were regarded as significantly enriched gene sets when P < 0.05, as well as KEGG signaling pathways.

### Protein-Protein Interaction (PPI) Network Construction

Based on the Search Tool for the Retrieval of Interacting Genes (STRING) database (https://string-db.org/) (32, 33), a PPI network of hub genes in the modules was constructed with a confidence > 0.4, maximum number of interactors = 0. Based on a tool called network analyzer in Cytoscape (34), the degree of connectivity of each hub gene was further calculated. Nodes in the PPI network represented proteins and edges represented protein-protein associations. Node color and node size were changed with degree of hub gene. Edge color and width were changed with combined score. Genes with the top 10 degree of connectivity were selected for further analysis. In addition, we used the MCODE plug-in in Cytoscape software to screen out hub modules in the PPI network, by using the following criteria: degree cutoff = 2, haircut on, node score cutoff = 0.2, k-core = 2, and max. depth = 100. Gene with the top 10 MCODE score were selected for further analysis. Furthermore, we used the cytoHubba plug-in in Cytoscape software to identify hub genes in the PPI network, the top 10 hub genes were screened out by using the Betweenness algorithm, maximum clique centrality (MCC) algorithm, and stress algorithm, respectively. After finishing these steps, genes that overlapped in the five methods were regarded as hub genes in the PPI network.

### Identification of Immune-Related Prognostic Biomarkers

To explore the prognostic value of hub genes in the PPI network, we further performed overall survival (OS) analysis by using GEPIA (http://gepia.cancer-pku.cn/) (35), an online tool based on TCGA data. Hub genes with significant *P* value in this analysis were considered as immune-related prognostic biomarkers in the present study. In addition, disease-free survival (DFS) analysis was also performed. Furthermore, a stage plot (I, II, III, and IV) was also drawn, the statistical significance of which was measured by one-way analysis of variance (ANOVA) test.

### Patients and Preparation of Specimens

After the surgery, a total of 20 samples, including 10 human BC tissues and 10 adjacent normal bladder tissues, were gathered from patients at Zhongnan Hospital of Wuhan University. The samples were histopathologically confirmed by two pathologists independently. The inclusion criteria are as follows (1): the histopathological type is confirmed as bladder urothelial carcinoma (BLCA) (2), not received anti-cancer treatment before cystectomy (3), underwent radical cystectomy or partial nephrectomy (4), no history of other malignant tumors. Exclusion criteria are as follows (1): other pathological types of BC (2), metastatic BLCA or other merge tumors (3), patients who did not undergo surgery, and (4) clinical pathological data are incomplete. Each patient signed an informed consent form, and the medical ethics committee in this hospital approved the utilization of tumor tissues for the present study. The approval number for this study was 2020174 (Kelun).

### Immune-Related Prognostic Biomarker Validation

To validate the prognostic value of immune-related prognostic biomarker, we performed survival analysis for hub gene based on GSE13507 and GSE32548 by R package “survival” (36). BC samples were divided into low-expression group and high-expression group in the two data sets, respectively. Then, OS using GSE32548 and cancer-specific survival (CSS) analysis using GSE13507 were performed. A *P* value less than 0.05 was considered significant. Furthermore, we assessed the mRNA expression levels of hub genes in BC and normal tissue by Oncomine database (https://www.oncomine.org/) (37). Two independent data sets were used in the present study. In addition, we performed quantitative real-time PCR (qRT-PCR) analysis. The expression patterns of the CD86 genes were evaluated in BCs and adjacent normal bladder tissues. The HiPure Total RNA Mini Kit and RNAiso-Plus (TAKARA, China) were used to extract total RNA from the cells and 10 pairs of bladder cancer tissue and adjacent normal bladder tissues, which collected from the Zhongnan Hospital of Wuhan University, and we used NanoDrop to quantify the RNA, which was then reverse transcribed into cDNA by ReverTra Ace qPCR RT Kit (Toyobo, China). Finally, we performed qRT-PCR analysis of cDNA with iQTM SYBR^®^ Green Supermix (Bio-Rad) in a final volume of 20 μl. Relative gene expression was quantified *via* the 2^−△△Ct^ approach and normalized to glyceraldehyde-3-phosphate dehydrogenase (GAPDH) expression. The primer sequences for CD86 molecule (CD86) and GAPDH were listed in **Table 1**. We measured the statistical significance by conducting Student *t* test. Finally, we validated the translation-level expression of hub gene between normal urinary bladder tissue and bladder urothelial carcinoma tissue by using The Human Protein Atlas (HPA) database (https://www.proteinatlas.org/) (38).

**Table 1:** List of primers for qRT-PCR.

Gene	Symbol	Forward primer (5′-3′)	Reverse primer (5′-3′)	Annealing temperature, C°)
CD86 molecule	CD86	5′-AGCCTTATCGGAAATGATCCAGT-3′	5′-GGCCTTGTAGACACCTTGGT-3′	60
Glyceraldehyde-3-phosphate dehydrogenase	GAPDH	5′-ACAACTTTGGTATCGTGGAAGG-3′	5′-GCCATCACGCCACAGTTTC-3′	60

CD86, CD86 molecule; GAPDH, glyceraldehyde-3-phosphate dehydrogenase; qRT-PCR, quantitative real time-polymerase chain reaction.

### Association Between Immune-Related Prognostic Biomarker Expression and Immunocytes Exploring

Immunocytes have been proven to be independent predictors of survival in cancers, thus, in this study, we investigated the relationship between expression levels of selected IRGS and immunocytes based on tumor immune estimation resource (TIMER) (https://cistrome.shinyapps.io/timer/). TIMER was a webtool, which could estimate the abundance of immune infiltrates for six tumor-infiltrating immune cell types. We thought an immune-related prognostic biomarker is strongly related to an infiltrating level of an immune cell type when |correlation coefficient (cor) |is 0.2 or greater and a P value is less than 0.05.

### Exploration of Immune Cell Infiltration

In this part, we first calculated tumor purity, immune score, and stromal score for each BC sample collected from TCGA-BLCA data (n = 408) by applying estimation of stromal and immune cells in malignant tumors using expression data (ESTIMATE) algorithm (by using “estimate” package in R software) (39). Considering that immune cells played important roles in the tumor microenvironment (TME) (40, 41). Relying on ssGSEA (a R package in R software) (42, 43), the relative infiltration of 28 kinds of immunocytes was quantified. A gene list of metagenes, which contained feature genes symbolizing for each immunocyte type, was retrieved from an article in Cell Reports (44). In ssGSEA, we calculated enrichment score for each immunocyte type, which represented the relative abundance of immunocyte. Zero was the minimal meanwhile one was the maximal score. A heatmap was further composed for visualization of the relative abundance of all kinds of immune cell types. Finally, we plotted an MDS plot and constructed a Gaussian fitting model for estimation of the bio-similarity of the immune cell filtration.

### Gene Set Enrichment Analysis (GSEA)

In this study, we performed GSEA (45) based on TCGA-BLCA data from TCGA database to explore the potential functions of immune-related prognostic biomarkers. First, 408 BCs were divided into low expression group (n = 204), and high expression group (n = 204) by setting the median of CD86 expression as a cut line. “c2.cp.kegg.v7.0.symbols.gmt” was set as the reference gene set. In this study, we thought KEGG signaling pathways were significantly enriched when nominal *P* value is less than 0.05, |enrichment score (ES)| is greater than 0.6, gene size is 100 or greater, and false discovery rate (FDR) is less than 25%.

## Results

### 1,333 IRGs Were Included for Co-Expression Network Construction

A comprehensive list of 2,498 IRGs was first downloaded from ImmPort database. Then, 1,333 genes overlapped between IRGs and genes in GSE32548 were analyzed and further included for WGCNA, the information of the 1,333 genes is shown in detail in **Table S1**.

### Two Key Modules Related to Pathological Stage Were Screened Out

**Figure 2:**
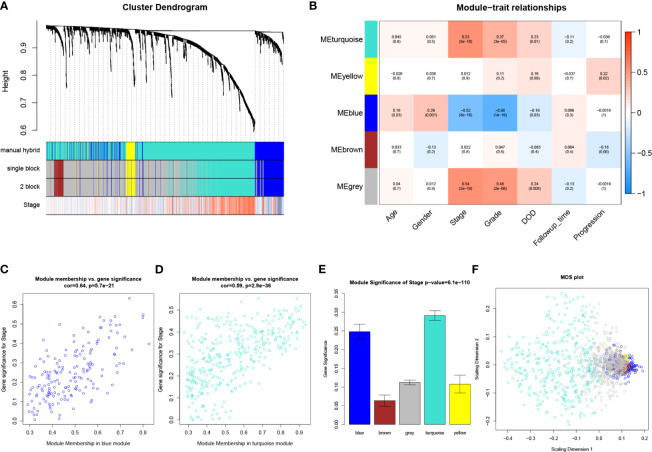
WGCNA analysis. **(A)** The cluster dendrogram of genes in GSE32548. Each branch in the figure represents one gene, and every color below represents one co-expression module. **(B)** Heatmap of the correlation between module eigengenes (MEs) and different clinical information of BC (age, gender, pathological stage, grade, DOD (death of disease), follow-up time, and progression). **(C)** Scatter plot of module eigengenes in the blue module. **(D)** Scatter plot of module eigengenes in the turquoise module. **(E)** Distribution of average gene significances and errors in the modules associated with the pathological stage of BC. **(F)** Classical MDS plot whose input is the TOM dissimilarity. Each dot (gene) is colored by the module assignment.

Six outliers were first removed from the expression profile of the 1,333 IRGs by using two independent methods (**Figures S1A, B**), only 122 samples (with complete clinical information) from GSE32548, which reached the standards, were included for subsequent analysis. As shown in **Figures S1C–F**, beta (β) = 2 (scale free R^2^ = 0.95) was further chosen for adjacency calculation. Then, we classified genes into gene modules. Moreover, we merged modules highly correlated to each other (pairwise correlation of modules > 0.75). In total, four modules including turquoise (n = 372), yellow (n =52), blue (n = 170), and brown (n = 55) were screened out (**Figure 2A**). The rest of the IRGs, including 684 genes, showed weak correlation with other genes, which were excluded for next-step analysis (these genes were discarded to gray module). Then, we noticed that the turquoise module showed the most positive association with pathological stage (*P* = 5E-10, R^2^ = 0.53), meanwhile the blue module was the most negatively associated module with pathological stage compared with others (*P* = 8E-10, R^2^ = −0.52), as suggested in **Figure 2B**. The association between MM and GS in the two modules is shown in **Figures 2C, D**, separately. MM in blue module was significantly associated with GS in blue module (cor = 0.64, *P* = 5.7E-21), a similar trend also existed in turquoise module (cor = 0.59, *P* = 2.9E-36). Moreover, the MS of the two modules was significantly higher than that of any other modules (**Figure 2E**). Therefore, blue module and turquoise module were screened out and regarded as key modules in the present study. As shown in **Figure 2F**, the MDS plot demonstrated that each module was isolated from each other, especially the key modules.

### Identification of 45 Hub Genes in Key Modules

In this study, 43 genes with |cor.geneModuleMembership| > 0.80 and |cor.geneTraitSignificance| > 0.20 were considered as hub genes in the turquoise module, meanwhile two genes that reached the same standards were regarded as hub genes in the blue module. The detailed information of these hub genes are shown in **Table S4**.

### Exploration of Function and Pathway of Hub Genes

**Figure 3:**
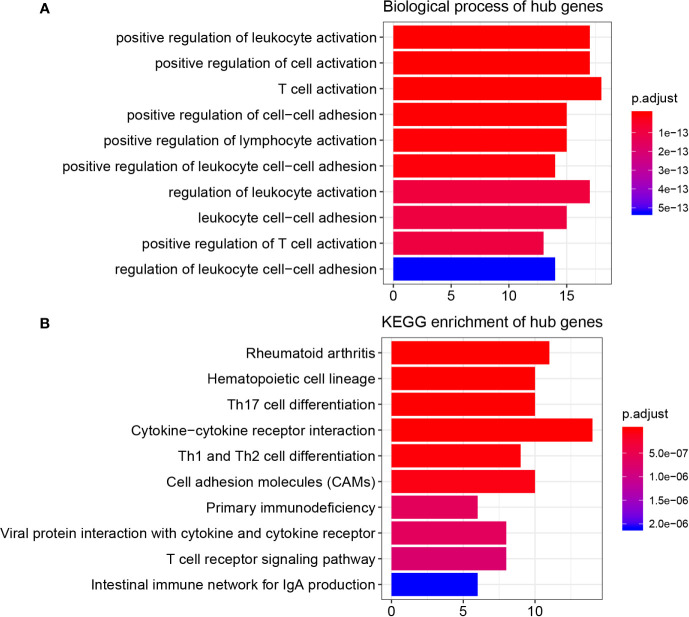
Function enrichment analysis, PPI network, and GEPIA. **(A)** GO analysis of hub genes in key modules. **(B)** KEGG pathway enrichment of hub genes in key modules.

As **Table S2** shows, the 45 hub genes were significantly enriched in 361 biological processes (BPs). The top 10 enriched BPs were positive regulation of leukocyte activation, positive regulation of cell activation, T cell activation, positive regulation of cell-cell adhesion, positive regulation of lymphocyte activation, positive regulation of leukocyte cell-cell adhesion, regulation of leukocyte activation, leukocyte cell-cell adhesion, positive regulation of T cell activation, and regulation of leukocyte cell-cell adhesion (**Figure 3A**). Furthermore, these hub genes were significantly related to 38 KEGG signaling pathways (**Table S3**). The top 10 KEGG pathways were rheumatoid arthritis, hematopoietic cell lineage, Th17 cell differentiation, cytokine-cytokine receptor interaction, Th1 and Th2 cell differentiation, cell adhesion molecules (CAMs), primary immunodeficiency, viral protein interaction with cytokine and cytokine receptor, T cell receptor signaling pathway, and intestinal immune network for IgA production (**Figure 3B**).

### PPI Network Construction and Immune-Related Prognostic Biomarker Identification

**Figure 4:**
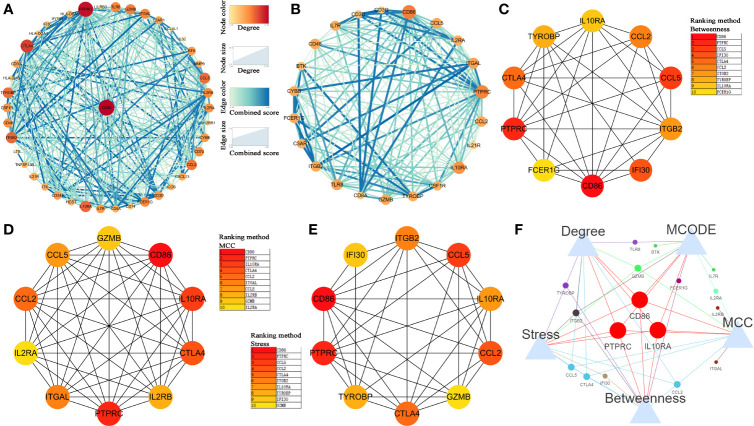
Identification of hub genes. **(A)** Protein-protein interaction (PPI) network of hub genes in key modules. **(B)** Top 1 module *via* MCODE. **(C)** The network of top 10 hub genes screened by CytoHubba Betweenness algorithm. **(D)** The network of top 10 hub genes screened by CytoHubba MCC algorithm. **(E)** The network of top 10 hub genes screened by CytoHubba Stress algorithm. **(F)** Venn diagram to screen out overlapped hub genes.

**Figure 5:**
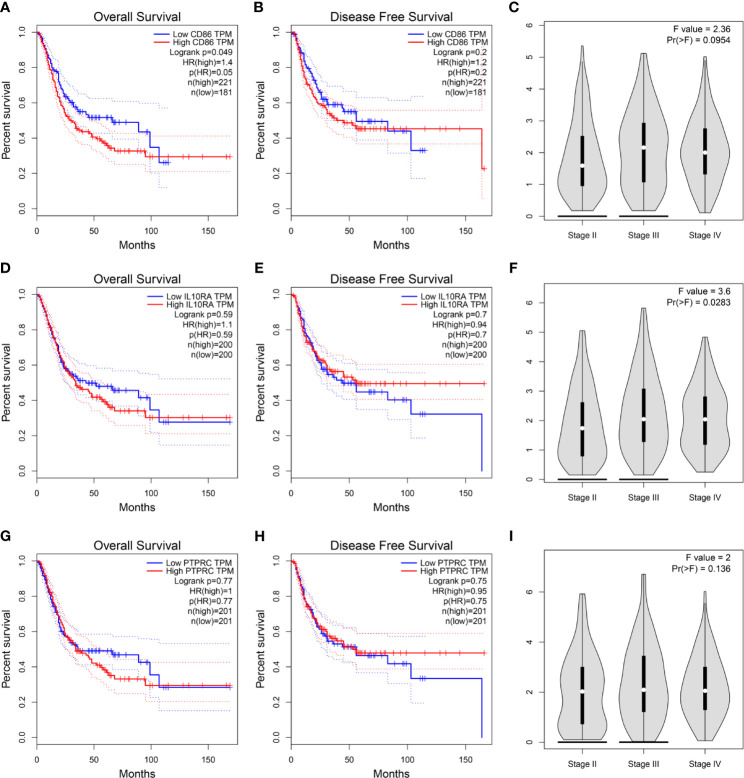
**(A)** Survival analysis of the association between the CD86 expression level and overall survival time in BC (based on GEPIA). **(B)** Survival analysis of the association between the CD86 expression level and disease-free survival time in BC (based on GEPIA). **(C)** stage plot of CD86 by using GEPIA. **(D)** Survival analysis of the association between the IL10RA expression level and overall survival time in BC (based on GEPIA). **(E)** Survival analysis of the association between the IL10RA expression level and disease-free survival time in BC (based on GEPIA). **(F)** stage plot of IL10RA by using GEPIA. **(G)** Survival analysis of the association between the PTPRC expression level and overall survival time in BC (based on GEPIA). **(H)** Survival analysis of the association between the PTPRC expression level and disease-free survival time in BC (based on GEPIA). **(I)** stage plot of PTPRC by using GEPIA.

Then, we constructed a PPI network for the 45 hub genes identified before (**Figure 4A**). The degree of connectivity of each gene was calculated, which is shown in detail in **Table S4**. Genes with the top 10 degree of connectivity were selected, including CD86, PTPRC, CTLA4, IL10RA, ITGB2, CCL5, TYROBP, CCL2, GZMB, and TLR8. Moreover, the MCODE plug-in revealed one important functional module in the PPI network (**Figure 4B**). Genes with the top 10 MCODE score were identified, including CD86, IL10RA, ITGB2, PTPRC, IL2RA, GZMB, IL7R, BTK, FCER1G, and TLR8. Then, 10 genes, including CD86, PTPRC, CCL5, IFI30, CTLA4, CCL2, ITGB2, TYROBP, IL10RA, and FCER1G, were screened out by using betweenness algorithm applied by cytoHubba plug-in (**Figure 4C**). Meanwhile, 10 genes, including CD86, PTPRC, IL10RA, CTLA4, CCL2, ITGAL, CCL5, IL2RB, GZMB, and IL2RA, were identified by MCC algorithm (**Figure 4D**). In addition, 10 genes, including CD86, PTPRC, CCL5, CCL2, CTLA4, ITGB2, IL10RA, TYROBP, and IFI30, were screened out by applying stress algorithm (**Figure 4E**). Finally, three genes, including CD86 molecule (CD86), IL10RA (interleukin 10 receptor subunit alpha), and protein tyrosine phosphatase receptor type C (PTPRC), overlapped among genes selected by the five methods were picked out (**Figure 4F**). We regarded the three genes as hub genes in the PPI network. We immediately performed OS analysis for the three genes, the result demonstrated that expression of CD86 could impact the survival and prognosis of patients with BC. Patients with high CD86 expression had worse OS (**Figure 5A**, *P* = 0.049), meanwhile there was a trend that high expression of CD86 caused worse DFS compared with low expression (**Figure 5B**, *P* = 0.2). A stage plot was shown in **Figure 5C**, and CD86 presented different expression levels in different stage (II, III, and IV). As for IL10RA (**Figures 5D, E** **)** and PTPRC (**Figures 5G, H** **)**, there was no obvious association between their expressions and survival. Stage plots were also plotted as shown in **Figures 5F, I**. Thus, only CD86 was considered as immune-related prognostic biomarker in the present study.

### Validation of CD86

**Figure 6:**
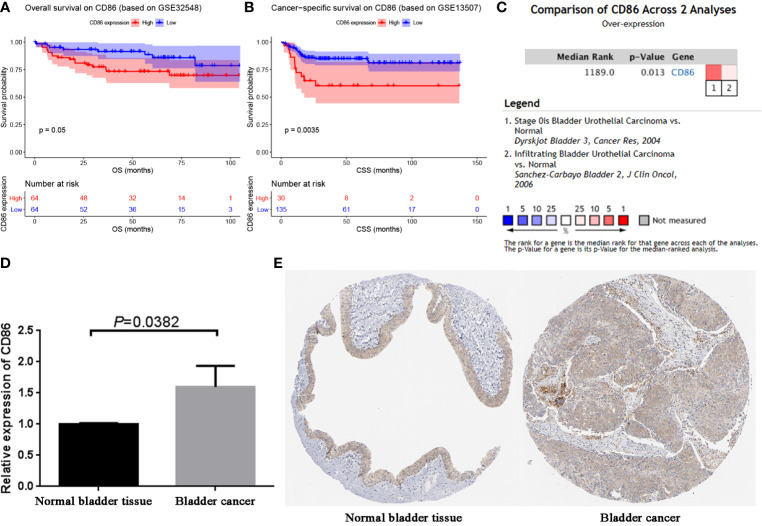
Validation of CD86. **(A)** Survival analysis of the association between the CD86 expression level and overall survival time in BC (based on GSE32548). **(B)** Survival analysis of the association between the CD86 expression level and cancer-specific survival time in BC (based on GSE32548). **(C)** Comparison of CD86 mRNA expression across 2 analyses of BC. **(D)** qRT-PCR analysis exhibited the expression of CD86 in bladder cancer tissues compared with the paired paracancerous tissues. **(E)** Validation of CD86 in translational level by The Human Protein Atlas database (IHC).

Based on GSE32548 and GSE13507, we validated the prognostic value of CD86. The result demonstrated that BC patients with high expression of CD86 had worse OS compared with these with low expression of CD86 (**Figure 6A**, *P* = 0.05). Also, high expression of CD86 in BC patients was significantly associated with worse CSS, as shown in **Figure 6B**. These results were consistent with what we found by using GEPIA. Then based on BC data from Oncomine database, we compared the expression of CD86 between BC tissues and normal bladder tissues. The result suggested that the CD86 mRNA expression was higher in BCs compared to normal tissues (*P* = 0.013, **Figure 6C**). We further validated the mRNA expression of CD86 by using qRT-PCR analysis again. As shown in **Figure 6D**, CD86 was significantly up-regulated in BC tissue compared with adjacent normal bladder tissue (*P* = 0.0382), which was consistent with the findings based on Oncomine database. By using HPA database, we also explored the translational-level expression of CD86, but not as we imagined, there was no significant difference between the expression in normal bladder tissue and bladder cancer tissue (**Figure 6E**).

### Correlation of CD86 Expression With Immune Infiltration Level in BC

**Figure 7:**
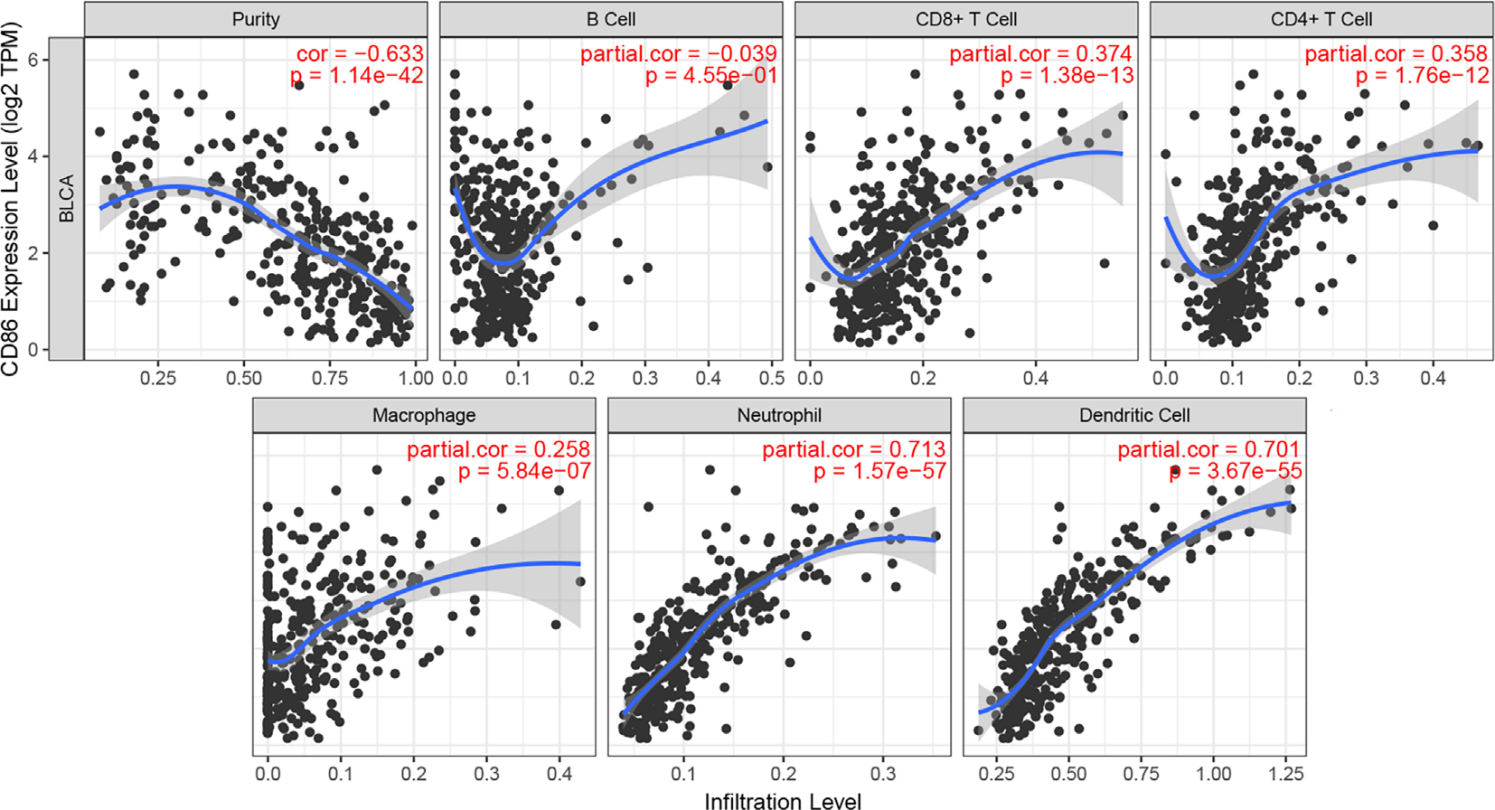
Correlation of CD86 expression with immune infiltration level in BC.

Immune infiltration was reported to be associated with survival and progression of cancers. Thus, by using TIMER (a webtool), the association between CD86 and immune infiltration level was obtained. As shown in **Figure 7**, CD86 was positively associated with CD8+ T cells (cor = 0.374, *P* = 1.38E-13), CD4+ T cells (cor = 0.358, *P* = 1.76E-12), macrophages (cor = 0.258, *P* = 5.84E-07), neutrophils (cor = 0.713, *P* = 1.57E-57), and dendritic cells (cor = 0.701, *P* = 3.67E-55).

### Immune Cell Infiltration

**Figure 8:**
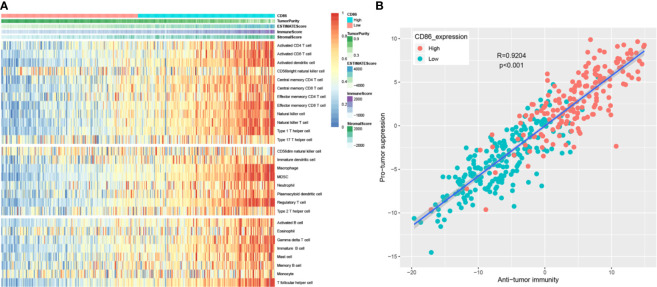
Correlation between CD86 expression and immune cell infiltration heterogeneity. Differentially expressed genes (DEGs) analysis in ccRCC. **(A)** Single-sample gene set enrichment analysis identifying the relative infiltration of immune cell populations for 408 BC tumor samples with available RNA-sequencing data. The relative infiltration of each cell type is normalized into a z-score. **(B)** Correlation between infiltration of cell types executing anti-tumor immunity (ActCD4, ActCD8, TcmCD4, TcmCD8, TemCD4, TemCD8, Th1, Th17, ActDC, CD56briNK, NK, NKT) and cell types executing pro-tumor, immune suppressive functions (Treg, Th2, CD56dimNK, imDC, TAM, MDSC, neutrophil, and pDC). R coefficient of Pearson’s correlation.

After calculating immune score and stromal score of each BC from TCGA-BLCA data, immune scores ranged from −1900.04 to 2903.20 meanwhile stomal scores ranged from −2496.63 to 2148.31 as the result suggested (**Table S5**). As shown in **Figure 8A**, the heatmap demonstrated that CD86 expression was significantly associated with ESTIMATE score, immune score, and stromal score, positively; meanwhile, it negatively correlated to tumor purity, which was consistent with the TIMER analysis. Furthermore, CD86 high-expression samples were associated with a higher abundance of immune cell infiltration. The ssGSEA enrichment scores (after normalization) for each immune cell type in 408 BC patients are shown in detail in **Table S6**. More concretely, high CD86 expression of patients was related to a higher abundance of immune cells executing anti-tumor reactivity, including activated CD4^+^ T cells, activated CD8^+^ T cells, activated dendritic cells, central memory CD4^+^ T cells, central memory CD8^+^ T cells, effector memory CD4^+^ T cells, effector memory CD8^+^ T cells, natural killer cells, natural killer T cells, and type 1 T helper cells. The abundances of immune cells, which delivered pro-tumor suppression (including macrophages, myeloid-derived suppressor cells (MDSCs), and regulatory T cells), were positively associated with CD86 expressions in patients with BC. Further analysis suggested that immune cells executing anti-tumor reactivity were positively related to immune cells delivering pro-tumor suppression within a local environment, significantly (**Figure 8B**, cor = 0.9204, *P* < 0.001). Interestingly, these results were consistent with what we got by TIMER, which reflected that anti-tumor inflammation might facilitated the recruitment or differentiation of cells specialized for immune suppression in BC.

### CD86-Related KEGG Signaling Pathways

To explore the potential function of CD86, we performed GSEA. As shown in **Table 2**, the result suggested that the high expression of CD86 was significantly correlated to nine KEGG signaling pathways, including systemic lupus erythematosus, cytokine-cytokine receptor interaction, cell adhesion molecules (CAMs), toll-like receptor signaling pathway, chemokine signaling pathway, natural killer cell-mediated cytotoxicity, T-cell receptor signaling pathway, focal adhesion, and leukocyte transendothelial migration.

**Table 2:** Genet set enrichment analysis (GSEA) in CD86 high-expression phenotype.

NAME	SIZE	ES	NES	NOM p value	FDR
KEGG_SYSTEMIC_LUPUS_ERYTHEMATOSUS	130	−0.76619	−1.75486	0	0.057827
KEGG_CYTOKINE_CYTOKINE_RECEPTOR_INTERACTION	257	−0.76283	−1.71835	0	0.048408
KEGG_CELL_ADHESION_MOLECULES_CAMS	128	−0.75899	−1.66159	0	0.058227
KEGG_TOLL_LIKE_RECEPTOR_SIGNALING_PATHWAY	101	−0.74039	−1.8011	0	0.143614
KEGG_CHEMOKINE_SIGNALING_PATHWAY	185	−0.73645	−1.77893	0	0.089826
KEGG_NATURAL_KILLER_CELL_MEDIATED_CYTOTOXICITY	131	−0.71724	−1.75548	0	0.065954
KEGG_T_CELL_RECEPTOR_SIGNALING_PATHWAY	108	−0.6936	−1.77886	0.002024	0.071861
KEGG_FOCAL_ADHESION	194	−0.6713	−1.69605	0.001988	0.05191
KEGG_LEUKOCYTE_TRANSENDOTHELIAL_MIGRATION	113	−0.65567	−1.72305	0.001927	0.049928

### Suloctidil Might Be a Novel Drug to Treat BC

Also, based on GSEA, we downloaded “DSigDBv1.0.gmt” from Drug SIGnatures Database (http://tanlab.ucdenver.edu/DSigDB/DSigDBv1.0/download.html) (46) and attempted to screen out some novel drugs for BC treatment. The result demonstrated that CD86 was associated with various drugs (**Table S7**). Among them, *suloctidil* with the highest |ES| was further screened out (nominal *P* = 0.000, |ES| = 0.824, gene size = 137, and FDR = 6.345%), which showed powerful potential to treat BC.

## Discussion

As the most common urinary malignancy of the urinary system, most patients with BC have to face poor prognosis. Nowadays, immunotherapy has become a novel approach for tumor treatment, which mainly uses the immune effects of autoimmune or alloimmune cells in patients to improve the symptoms, prolong the survival, and improve the prognosis (47, 48). Therefore, we aimed to identify some novel prognostic biomarkers, which were associated with immune microenvironment in BC.

In the present study, some bioinformatics methods were used to explore immune-related prognostic biomarker of BC. After conducting WGCNA, a total of 45 potential hub genes were identified. Lu et al. used CytoHubba MCC method in their study to screen out hub genes, whereas Huang et al. identified serpin family E member 1 (SERPINE1) as a novel biomarker for diffuse lower-grade gliomas *via* CytoHubba stress algorithm and CytoHubba betweenness algorithm (49, 50). Considering that all these algorithms were effective methods to screen out hub genes, we screened out three hub genes among the 45 genes by using all the algorithms in the present study to make our results credible. CD86 molecule (CD86) was further determined to show a strong association with the prognosis of BC by performing survival analysis *via* three independent data sets. Thus, CD86 was regarded as an immune-related prognostic biomarker of BC. There were also some previous studies showing the association of CD86 with bladder cancer. Recent studies demonstrated that direct augmentation of BC immunogenicity offered a potential therapeutic strategy for BC (51). The clinical response to bacille Calmette-Guerin (BCG) therapy might be improved by concurrent enhancement of tumor immunogenicity (51). Pettit et al. demonstrated that BC line J82 could be transfected to functionally express the costimulatory molecules CD80 and CD86 (51). After IFN-g stimulation, J82 cells also express levels of MHC antigens and adhesion molecules, which can activate antigen-specific T cells efficiently (51). These results demonstrated that CD86 might be essential for BC therapy (51). In addition, Goux et al. found that low overexpression of CD86 in non–muscle-invasive bladder cancer (NMIBC) tissue, with no significant difference in mRNA expression as compared with normal bladder tissue, which suggested a minor role for the immune checkpoints in the early stages of bladder carcinogenesis (52). Further analysis suggested that CD86 was overexpressed in muscle-invasive bladder cancer (MIBC) compared with normal bladder tissue, which indicated that CD86 might be a marker of aggressiveness during urothelial carcinogenesis (52). As a limitation, both the studies did not explore the association of CD86 with BC prognosis. In our study, after using bioinformatics methods to screen out CD86 *via* strict thresholds for each step, we focused on exploring the prognostic value of CD86 in bladder cancer. We first performed overall survival analysis *via* GEPIA, the result indicated that BC patients with high expression of CD86 had worse OS compared with those with low expression of CD86. To improve the credibility of the result and avoid the contingency of the result, we performed survival analysis *via* two independent data sets, the results demonstrated that CD86 was a powerful prognostic biomarker for patients with BC. In addition, we also validated CD86 expression in mRNA-level (Oncomine database, qRT-PCR) and translational-level (HPA database), the result demonstrated that CD86 was overexpressed in BC, which was consistent with what Goux et al. did in their study.

Because of the important effect of immune infiltration level in survival in tumors, we explored the association between CD86 expression and immune infiltration of 28 immune cell types. High expression of CD86 was significantly correlated to higher abundances of various immune cells, including two major types, cells that executed anti-tumor reactivity: activated CD4+ T cells, activated CD8+ T cells and cells that delivered pro-tumor suppression: macrophages, myeloid-derived suppressor cells (MDSCs), and regulatory T cells. In summary, we found that anti-tumor inflammation might facilitate the recruitment or differentiation of cells specialized for immune suppression in BC.

Moreover, we found that a drug named *suloctidil* might be a novel choice for BC treatment. *Suloctidil* was a vasodilator and anti-platelet agent. Zeniou et al. indicated that *suloctidil* might be considered as a cytotoxic agent in a glioblastoma stem-like cells, with no specificity toward cancer cells at concentrations in the low micromolar range (53). Thus, more ambitious in-depth study must be done to explore the relationship of *suloctidil* with bladder cancer treatment.

Some limitations of the present study should be discussed. Although we designed this bioinformatic and experiment study well, some negative results existed. First, when we performed the DFS analysis based on GEPIA, the *P* value was more than 0.05, perhaps, because of the particularity of survival cutoff point (DFS rather than OS). Thus, we will perform DFS analysis by using larger data sets from public database or clinical collection. Second, the result of translation-level expression validation of CD86 based on HPA database was not as well as we expected. Thus, we will perform Western blotting (WB) analysis to validate the CD86 translation-level expression in our further research.

In conclusion, for the first time, we constructed co-expression network for IRGs in BC. CD86 was screened out and validated by using some bioinformatics methods and experimental assays based on the data sets from public databases and Zhongnan Hospital of Wuhan University, which were regarded as an immune-related prognostic biomarkers in BC. Moreover, a small drug named *suloctidil* might be a novel choice for clinicians to treat BC.

## Data Availability Statement

Publicly available data sets were analyzed in this study. The data sets generated and/or analyzed in the present study were retrieved from TCGA database (https://genome-cancer.ucsc.edu/) and GEO database (http://www.ncbi.nlm.nih.gov/geo/).

## Author Contributions

T-ZL, SL, and XY conceived and designed the study. G-WD, XY, and ZC performed the analysis procedures. G-WD, XY, ZC, and T-ZL analyzed the results. T-ZL, SL, and XY contributed analysis tools. G-WD and XY contributed to the writing of the manuscript. All authors reviewed the manuscript. All authors contributed to the article and approved the submitted version.

## Funding

This work was supported by the National Natural Science Foundation of China (81802541), the Science and Technology Department of Hubei Province Key Project (2018ACA159), and the Medical Science and Technology Innovation Platform Support Project of Zhongnan Hospital of Wuhan University (PTXM2019006).

## Conflict of Interest

The authors declare that the research was conducted in the absence of any commercial or financial relationships that could be construed as a potential conflict of interest.

## Publisher’s Note

All claims expressed in this article are solely those of the authors and do not necessarily represent those of their affiliated organizations, or those of the publisher, the editors and the reviewers. Any product that may be evaluated in this article, or claim that may be made by its manufacturer, is not guaranteed or endorsed by the publisher.
