# Supplementary figures and images for: CD86 Molecule Might Be a Novel Immune-Related Prognostic Biomarker for Patients With Bladder Cancer by Bioinformatics and Experimental Assays
Source: Front Oncol. 2021 Aug 6;11:679851. doi: 10.3389/fonc.2021.679851 (PMC8378807; doi:10.3389/fonc.2021.679851)

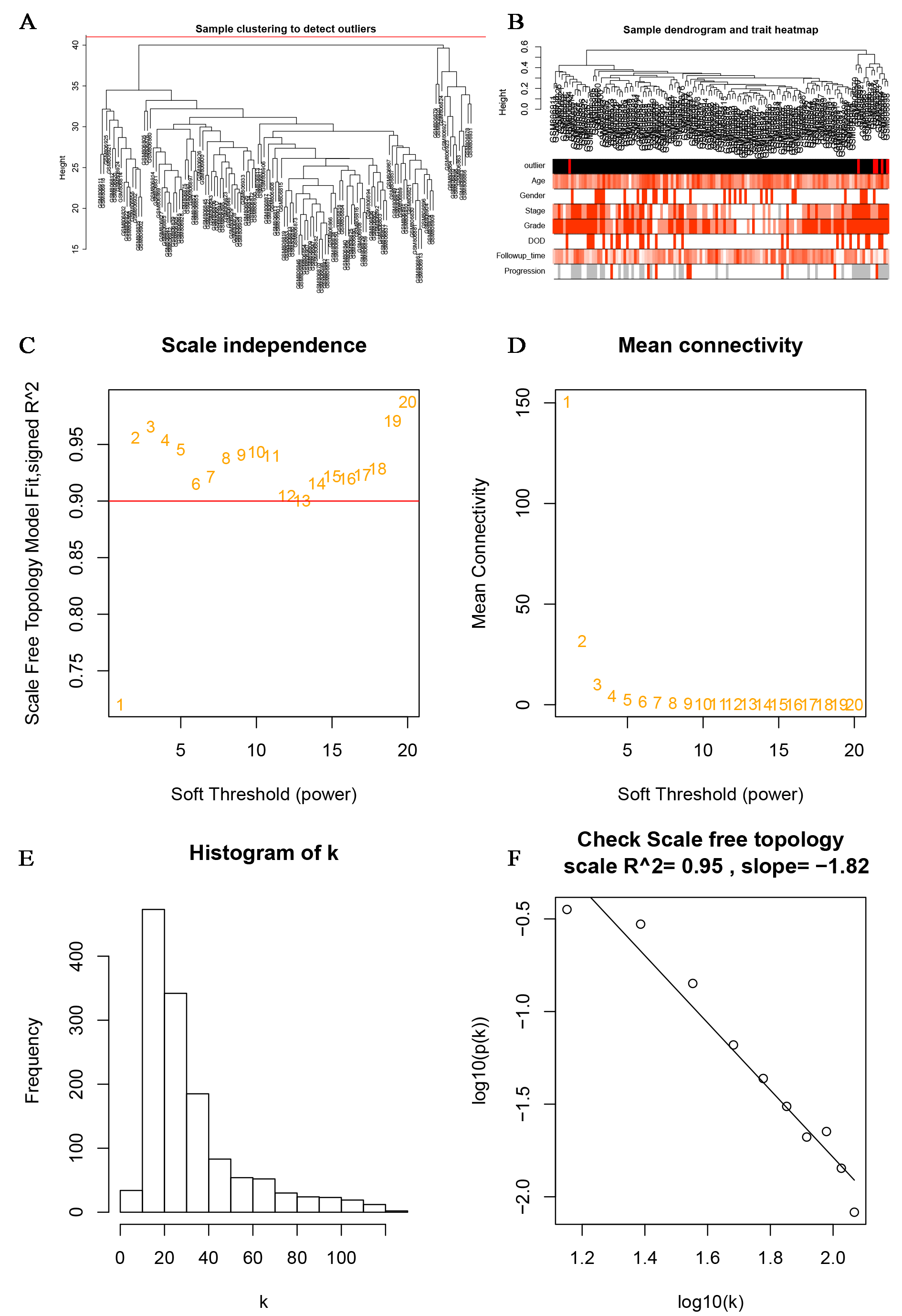

Supplement: Supplementary file 1 [file Image_1.tif]
